# In Vitro assessment of anti*-Campylobacter* activity of *lactobacillus* strains isolated from canine rectal swabs

**DOI:** 10.1186/s12917-022-03204-9

**Published:** 2022-03-22

**Authors:** Anna Tomusiak-Plebanek, Martyna Mruk, Sybilla Rząca, Magdalena Strus, Zbigniew Arent

**Affiliations:** 1 Chair of Microbiology, Jagiellonian University Medical College, 31-121 18 Czysta Street, Krakow, Poland; 2 University Centre of Veterinary Medicine, University of Agriculture, 30-059 Krakow, Poland

**Keywords:** *Campylobacter*, Dog, Antibacterial activity, *Lactobacillus*, Probiotics

## Abstract

**Background:**

Campylobacteriosis is currently the most frequently reported zoonosis. Dogs, especially puppies or those with diarrhea, are considered a possible source of human infection. Probiotic bacteria, such as *Lactobacillus* species, seem to be a valuable tool in controlling of intestinal pathogenic microorganisms in dogs. The main purpose of this study was to assess the anti-*Campylobacter* activity and some probiotic properties, like ability to produce H_2_O_2,_ bile salt and low pH tolerance of *Lactobacillus* strains isolated from gastrointestinal tract of healthy dogs.

**Results:**

A total of 39 rectal swabs derived from healthy dogs and 19 from dogs with diarrhea were examined to detect *Lactobacillus* and *Campylobacter* bacteria respectively. In total, 30 strains of *Lactobacillus* genus and four strains of *Campylobacter* genus were isolated and identified. Of the 30 strains of *Lactobacillus*, 22 showed an inhibitory effect towards *Campylobacter*. Four strains with the strongest antagonism towards *Campylobacter* bacteria (*L. salivarius* 25 K/L/1, *L. rhamnosus* 42 K/L/2, *L. sakei* 50 K/L/1 and *L. agilis* 55 K/L/1) were selected to assess their potential probiotic traits. Three out of four analyzed strains produced extracellular H_2_O_2._ All displayed very good or moderate survival at pH 3.0 and 2.0 and showed high tolerance to 0.5% and 1% bile salts.

**Conclusions:**

Among selected *Lactobacillus* strains, all may have a potential probiotic application in reducing *Campylobacter* spp. in dogs and thus prevent transmission of infection to humans, although the best candidate for probiotic seems to be *L. sakei* 50 K/L/1. Further in vitro and in vivo studies are needed.

## Background

*Campylobacter* is a Gram-negative, microaerophilic bacteria causing one of the most common bacterial gastroenteritis. This organism is frequently found in the alimentary tract of numerous host species, including companion animals. For humans, infections with *Campylobacter* species, most commonly *C. jejuni* and *C. coli,* are the main cause of food-borne diarrhea [1]. In 2019, in European Union, there were noted 220,682 cases of campylobacteriosis, while the numbers of *Salmonella* cases were 87,923 [2]. The most common sources of infections are consumption of raw and undercooked poultry, unpasteurized milk, contaminated water, and direct animal contact [3]. It is estimated (data from 2017) that, around 6% cases of human campylobacteriosis are caused by contact with pets [4]. In recent years, it has been demonstrated that dogs, especially those less than 6 months of age, should be regarded as a potential source of *Campylobacter* infections [5, 6]. Predominant *Campylobacter* species detected in feces of dogs are *C. upsaliensis, C. jejuni, C. helveticus* and *C. coli* [5, 7, 8].

Dogs and cats are generally considered as asymptomatic carriers of *Campylobacter*. However, this pathogen can cause severe, gastrointestinal disease, particularly in young or immune suppressed animals [9]. Unfortunately, the extended treatment of bacterial-associated diarrhea with broad-spectrum antibiotics can result in increased antimicrobial resistance [10]. Therefore, there is a need for alternative therapies, such as probiotics.

Bacteria of the genus *Lactobacillus* are recognized candidates for probiotics. They are non-pathogenic organisms that may eliminate unfavorable microflora by several mechanisms such as the production of antimicrobial substances (lactic acid, bacteriocins, hydrogen peroxide), inhibition of bacterial adhesion to the mucosa, competition for nutrients, and stimulation of immunity [11]. There are several studies investigating the antimicrobial activity and metabolic potentials of *Lactobacillus* species isolated from the intestinal tract of dogs [12–14]. Currently, most of the probiotic microorganisms widely used in application studies on dogs are mainly of human origin [15, 16]. Potential probiotic strains for dogs are preferred to be of canine intestinal origin, since such strains exhibit host specificity [17]. Therefore, the aim of this study was to isolate, identify and evaluate the anti-*Campylobacte*r activity of *Lactobacillus* spp. strains derived from the intestinal tract of healthy dogs, as well as to assess some probiotic traits of selected strains, like ability to produce H_2_O_2,_ and bile salt, and low pH tolerance. To our knowledge there are no published studies so far, which assess anti-*Campylobacter* activity of canine-originated *Lactobacillus* bacteria.

## Results

### Identification of *Lactobacillus *and *Campylobacter* strains

From 39 rectal swabs of healthy dogs, a total of 30 strains of the genus *Lactobacillus* were isolated, which was confirmed by genus-specific Polymerase Chain Reaction (PCR) analysis. Using API 50CH test and species-specific PCR assays, 28 isolates were classified into six species, i.e., *L. brevis* (*n* = 4), *L. casei* (n = 4), *L. crispatus* (*n* = 2), *L. delbrueckii* (*n* = 3), *L. rhamnosus* (*n* = 8) and *L. salivarius* (*n* = 7). In the case of two *Lactobacillus* isolates, species identification using previously mentioned methods was ambiguous, so these strains were classified by MALDI-TOF MS. One of them was classified as *L. sakei* and the other as *L. agilis* with a Biotyper log (score) values greater than 2.0 (high-confidence identification).

*Campylobacter* strains were isolated from 4 (21%) out of 19 dogs with diarrhea (one strain from each sample). By API Campy system and species-specific PCR technique three strains were identified as *C. jejuni* and one as *C. upsaliensis*.

### Agar slab method

**Fig. 1:**
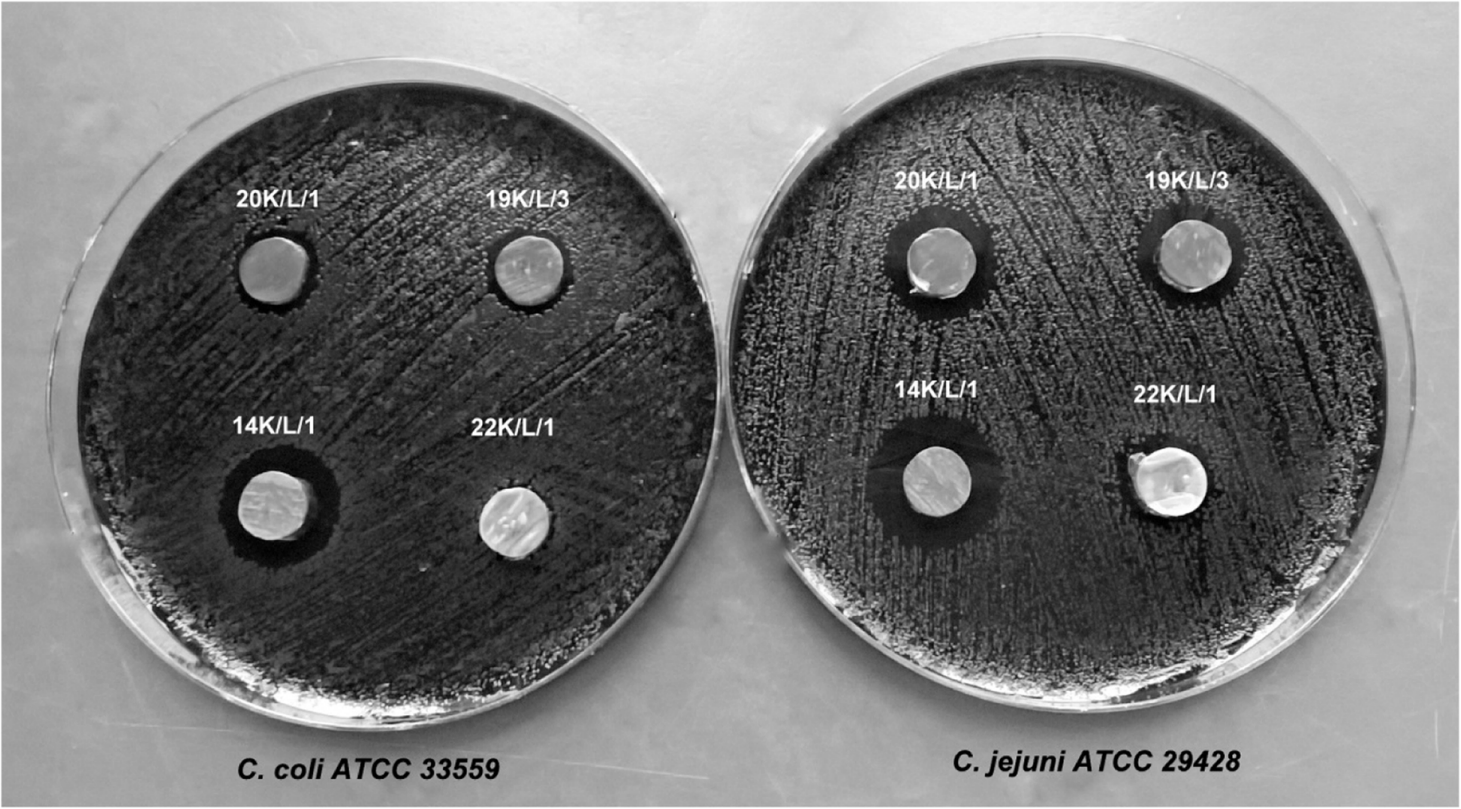
Antagonistic activity of some *Lactobacillus* isolates against reference strains of *Campylobacter* in the agar slab method

Results obtained by the agar slab method are presented in Table 1 as the mean diameter of the inhibition zone ± SD (standard deviation). Among 30 strains of *Lactobacillus*, 22 showed an inhibitory effect towards two reference *Campylobacter* strains (*C. jejuni* ATCC 29,428 and *C. coli* ATCC 33,559). The diameter of the growth inhibition zones induced by all *Lactobacillus* strains ranged from 9.0 ± 0.0 mm to 21.25 ± 1.1 mm, where 9 mm was the diameter of the slab (Fig. 1). The activity of the tested *Lactobacillus* strains against indicator bacteria was mainly correlated with the species. All analyzed isolates of *L. delbruecki*i (*n* = 3) exhibited no antagonistic properties (9.0 ± 0.0 mm) towards *C. jejuni* and *C. coli*, but strains of *L. rhamnosus* (*n* = 8) or *L. brevis* (*n* = 4) were all active against these pathogens. For strains belonging to the species *L. salivarius*, there was observed a large heterogeneity of the size of inhibitory zones. In this group, there was four out of seven strains that exhibited no antagonistic properties towards indicator strains and one isolate with the strongest inhibition of *Campylobacter* growth (mean inhibition zone 20.25 ± 1.4 mm).

**Table 1:**
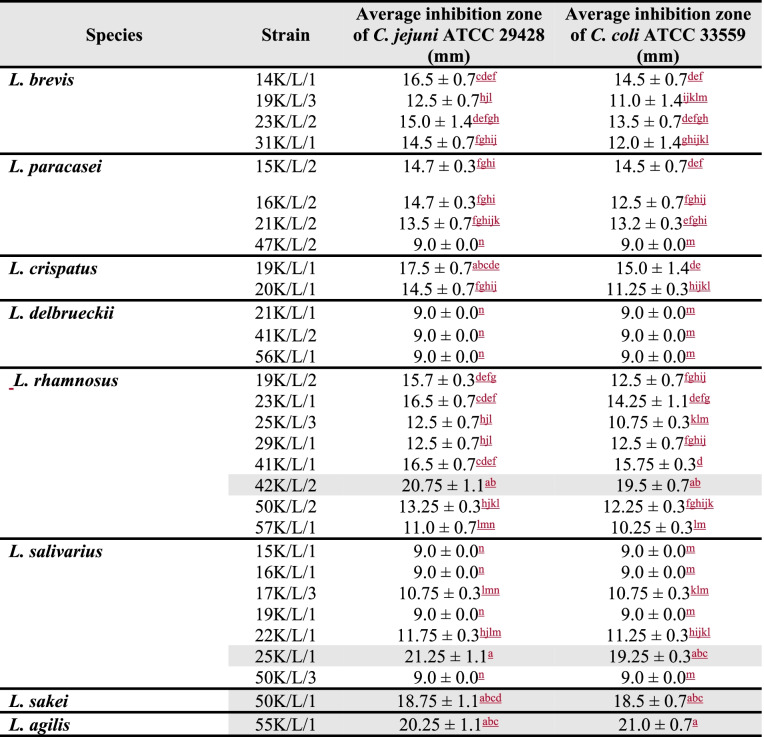
Anti-*Campylobacter* activity of *Lactobacillus* isolates

Results of agar slab method are presented as the mean diameter of the inhibition zone ± SD. Means with different letters within the same column indicate significant difference at *P* < 0.05

Grey highlights indicate strains that have been selected for further research

The average diameter of inhibition zones for *C. jejuni* was 13.6 ± 3.8 mm, and for *C. coli* 12.6 ± 3.4 mm. There were no statistically significant differences (P < 0.05) in susceptibility of these two species of *Campylobacter* to the antagonistic substances produced by *Lactobacillus* strains.

### Serial dilution method

**Fig. 2:**
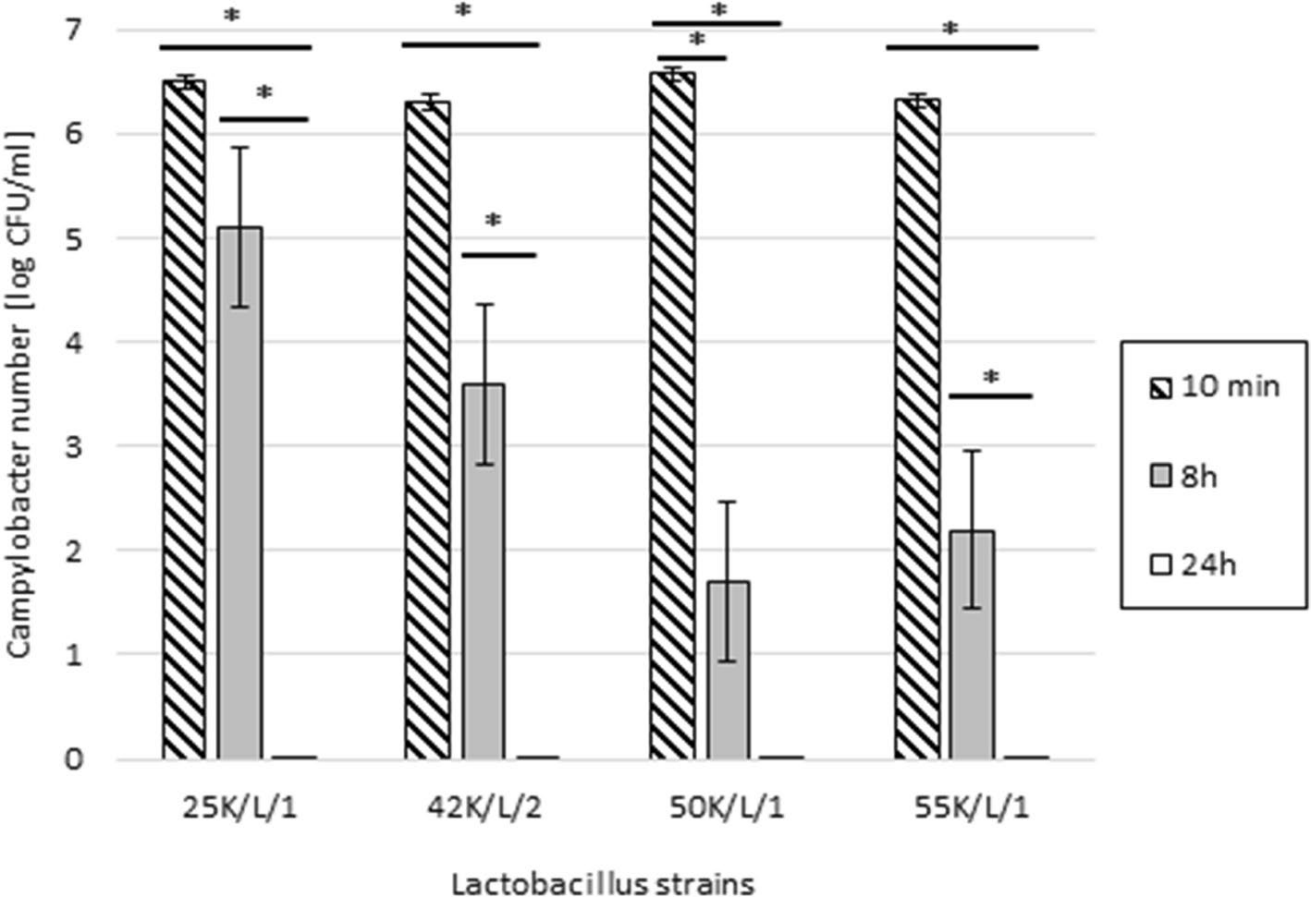
Relation of *Campylobacter* number [log CFU/ml] with the antibacterial activity of *Lactobacillus* strains. Error bars represent standard errors. Asterisks indicate the statistical differences: * *P* < 0.05

Among four selected *Lactobacillus* strains: 25 K/L/1 (*L. salivarius*), 42 K/L/2 (*L. rhamnosus*), 50 K/L/1 (*L. sakei*) and 55 K/L/1 (*L. agilis*), that had induced the biggest growth inhibition zones in the agar slab method, all confirmed strong inhibitory properties towards *Campylobacter* strains isolated from dogs. During the experiment, the *Campylobacter* number changed significantly (Fig. 2). After 8 h since the start of the procedure, the *Campylobacter* counts decreased by 1–5 logarithms (depending on the *Lactobacillus* strains). The most active strain turned out to be *L. sakei* 50 K/L/1. Only its activity contributed to a statistically significant reduction in the number of *Campylobacter* cells during the first 8 h of the experiment (*P* < 0.05). The remaining strains of *Lactobacillu*s also showed high antimicrobial activity. Within 24 h all resulted in a complete inhibition of the growth of *Campylobacter* bacteria.

### Well diffusion method

The pH values of native acidified supernatants obtained from 24 h cultures of *Lactobacillus* isolates ranged from 3.8 to 4.5. The average diameter of the growth inhibition zones induced by native acidified supernatants of tested 4 *Lactobacillus* strains was 16.26 ± 0.79 mm, where the well diameter was 9 mm. The average diameter of the growth inhibition zones induced by these strains in agar slab method was 19.9 ÷ 1.05. This difference is statistically significant (*P* < 0.05). Supernatants of *Lactobacillus* with neutralized acids (pH 6.8—7.0) did not exhibit any antagonistic activity towards *Campylobacter* bacteria.

#### Production of H_2_O_2_

Three out of four mentioned above strains of *Lactobacillus* produced extracellular H2O2. The highest rate of production (10 mg/L after 10 min from the start of the experiment and 30 mg/L 24 h later) was observed in 50 K/L/1 strain. Strains 25 K/L/1 and 55 K/L/1 after 24 h of incubation showed lower H2O2 production, 1 mg/L and 3 mg/L respectively. Detailed results are shown in Table 2.

**Table 2:** Production of H_2_O_2_ by selected *Lactobacillus* strains

Strain	Species	Ability to produce H2O2 (mg/L)		
		**10 min**	**4 h**	**24 h**
25 K/L/1	*L. salivarius*	1	1	1
42 K/L/2	*L. rhamnosus*	0	0	0
50 K/L/1	*L. sakei*	10	10	30
55 K/L/1	*L. agilis*	1	1	3

SD data were negligible

### Tolerance to low pH

All selected *Lactobacillus* strains were tolerant to acidic environment. The highest tolerance to low pH showed two strains: 50 K/L/1 (89.9% of viability at pH 3.0 and 69.1% at pH 2.0) and 42 K/L/2 (80.1% of viability at pH 3.0 and 79.5% at pH 2.0). Strains 25 K/L/1 and 55 K/L/1 had a good survival rate at pH 3.0 (percentage of the viability was 85.4 and 83.4% respectively) and a poorer survival rate at pH 2.0 (percentage of the viability was 50.1 and 28.6% respectively). Detailed results are shown in Table 3.

**Table 3:** The ability to survive of tested *Lactobacillus* strains in the different pH values

Strain	Ability to survive in low pH values				
	**pH 6.5 (control)**	**pH 3.0**		**pH 2.0**	
	**log (CFU/ml)**^**a**^	**log (CFU/ml)**^**a**^	**viability [%]**	**log (CFU/ml)**^**a**^	**viability [%]**
25 K/L/1	8.71 ± 0.03^b^	7.44 ± 0.57^a^	85.4	4.44 ± 0.33^c^	50.1
42 K/L/2	9.02 ± 0.03^a^	7.23 ± 0.16^a^	80.1	7.17 ± 0.18^a^	79.5
50 K/L/1	8.29 ± 0.06^c^	7.45 ± 0.16^a^	89.9	5.73 ± 0.17^b^	69.1
55 K/L/1	8.62 ± 0.05^b^	7.19 ± 0.16^a^	83.4	2.47 ± 0.21^d^	28.6

^**a**^Values are mean ± standard deviations, *N* = 2

Means with different letters within the same column indicate significant difference at *P < *0.05

### Bile salt tolerance

The tested isolates of lactic acid bacteria were exposed to bile salts in concentrations of 0.5% and 1%. All four *Lactobacillus* strains employed in the study were tolerant to both bile salts concentrations (percentage of the viability ranged from 78.2 to 88.8%). The highest resistance to bile was demonstrated by strain 50 K/L/1 (88.8% of viability at 0.5% bile salts concentration and 86.4% of viability at 1% bile salts concentration). Survival of all strains is presented in Table 4.

**Table 4:** The ability to survive of tested *Lactoabcillus* strains in the different concentration of bile salts

Strain	Ability to survive in the presence of bile salts				
	**MRS (control)**	**MRS + 0.5% bile**		**MRS + 1% bile**	
	**log CFU/ml**^**a**^	**log (CFU/ml)**^**a**^	**viability [%]**	**log (CFU/ml)**^**a**^	**viability [%]**
25 K/L/1	8.49 ± 0.41^abc^	6.94 ± 0.76^a^	81.7	6.64 ± 0.57^a^	78.2
42 K/L/2	8.74 ± 0.08^ab^	7.20 ± 0.12^a^	82.4	7.06 ± 0.16^a^	80.8
50 K/L/1	8.15 ± 0.15^c^	7.24 ± 0.17^a^	88.8	7.04 ± 0.15^a^	86.4
55 K/L/1	8.81 ± 0.12^a^	7.31 ± 0.23^a^	82.9	7.28 ± 0.28^a^	82.6

^a^Values are mean ± standard deviations, *N* = 2

Means with different letters within the same column indicate significant difference at *P* < 0.05

## Discussion

In recent years, there has been an increased interest in using probiotics in small animal veterinary medicine. In the literature only a few studies can be found, where probiotic strains have been isolated from dogs [17–19]. In this study, 30 strains of *Lactobacillus* spp. were isolated from rectal swabs of healthy dogs and assessed for their antagonistic activity against *Campylobacter* spp. strains.

The results of agar slab method showed that most of the tested *Lactobacillus* strains were able to inhibit the growth of *C. jejuni* and *C. coli*. To confirm the capacity to produce some antimicrobial substances by four selected strains, we analyzed the activity of cell-free broth using well diffusion and serial dilution methods and we assessed the ability to synthesize H_2_O_2_. The results of the serial dilution method indicated that all four *Lactobacillus* strains show strong inhibitory effect towards *Campylobacter* bacteria of canine origin: *C. upsaliensis* and *C. jejuni*, but the strongest was exhibited by *L. sakei* 50 K/L/1. The results of the well diffusion method indicated that the reduced pH of the supernatant (due to lactic acid) play a key role in inhibiting the growth of *Campylobacter* bacteria. Neal-McKinney et al. showed that production of antimicrobial substances, like lactic acid, by *Lactobacillus* strains is responsible for disrupting the membrane of *Campylobacter* and reducing the growth of these pathogens [20]. The pH-dependent, anti-*Campylobacter* activity of cell-free supernatants of *Lactobacillus* strains has also been demonstrated by Bratz et al. [21].

*L. sakei* 50 K/L/1 turned out to be not only the most active isolate, but also the only one, at which a relationship between antimicrobial effect and high production of hydrogen peroxide was observed. Hydrogen peroxide is a very potent, biologically active substance that react with lipids, proteins and nucleic acids causing oxidative cell damage [22]. Antimicrobial activity of H_2_O_2_-producing *Lactobacillus* has been already proven [23–25]. The quantity of H_2_O_2_ produced by different *Lactobacillus* species varies, depending on the strain and for some of them production is not observed [26]. Some results indicate that there is no correlation between antimicrobial activity and H_2_O_2_ production of strains [27, 28]. It has also been confirmed in this study. Strains other than *L. sakei* 50 K/L/1, exhibited strong inhibition of *Campylobacter* growth, but their production of H_2_O_2_ was generally weak. The lack of relationship between amounts of hydrogen peroxide production and the antimicrobial activity of *Lactobacillus* strains isolated from fecal microbiota of dogs has also been described previously [29]. It is suggested that synthesis of hydrogen peroxide is a rare feature of intestinal *Lactobacillus*, as it is mainly related to vaginal isolates [30].

To check the capacity of *Lactobacillus* strains to act as probiotics, it is essential to determine their ability to survive in the low pH and in the high concentration of bile salt that are present in the intestinal tract. Thus, we decided to check whether the chosen strains display these properties. The pH levels of gastric juice may vary from 2.5 to 3.0 depending on the kind and feeding time, the growing stage, as well as the kind of animal [31]. Therefore, the survival of *Lactobacillus* strains was tested in deMan-Rogosa-Sharpe (MRS) medium with pH adjusted to 2.0, 3.0 and 6.5 (optimal conditions). Bacterial growth under low pH was monitored for 3 h, as it simulates bacterial residency in the stomach [32]. The results on acid tolerance showed that all tested *Lactobacillus* strains were tolerant to acidic environment. All strains had a good survival rate at pH 3.0 (< 20% of inhibition), but a moderate survival rate at pH 2.0. The highest tolerance to pH 2.0 showed two strains 50 K/L/1 and 42 K/L/2. After 3 h of incubation at pH value 2.0, they exhibited respectively 69,1% and 79,5% of viability. The results described in other studies confirm that after 3 h of exposure to a pH ≤ 2, the viability count of lactic acid bacteria is significantly reduced [33–35]. According to Liong and Shah reports, the survival of *Lactobacillus* strains at pH 3.0 for 2–3 h is acceptable as one of the requirements for the bacteria to be considered as probiotics [36]. Although most bacteria survive poorly at low pH values, it is suggested, that bacteria of intestinal origin tend to be more resistant to gastric pH, what is in accordance with our results [37]. The results obtained in the current study suggest also, that the tolerance of low pH values, is primarily strain-dependent feature.

Resistance to bile is also very important feature for probiotic strains, which affects their survival and the ability to reach the large intestine. The mechanism of toxic depletion of bile salts onto bacterial cells is not fully understood. However, it is known that these are amphipathic molecules which act as detergents that damage cell walls demonstrating a strong antimicrobial activity [38]. There is little information regarding bile concentration in the canine intestine. In the literature, for similar studies, the bile concentration of 0.3% is most often used as the corresponding amount of this component in the human small intestine [31, 39]. However, the variability of this parameter is emphasized in relation to both humans and animals. Strompfova et al. checked the survival rate of *L. fermentum* AD1 of canine origin in the presence of 1% bile [40]. In turn, Coman et al. assessed the tolerance of probiotic strains isolated from dog faecal samples to bile salts at concentration 0.1%, 0.3% and 0.5% [12]. Based on the above literature data, *Lactobacillus* strains in the current study were treated with bile salts at a concentration 0.5% and 1%. All tested *Lactobacillus* strains were very tolerant to both concentrations of bile, but the best survival rate showed strain 50 K/L/1.

## Conclusions

This study showed that all four selected strains of *Lactobacillus* may have potential application in reducing the level of canine intestine colonization by *Campylobacter* spp. and thus prevent infections in both dogs and humans. All strains possess characteristics of a probiotic candidates, but the most promising seems to be *L. sakei* 50 K/L/1. This strain turned out to be the most active against *Campylobacter* bacteria and possessed the highest tolerance for bile salts and acidic pH. Moreover, it produces high concentrations of hydrogen peroxide, which is a desirable feature of probiotic bacteria and unique in strains that usually live in the intestine. Further experiments are needed to investigate antibiotic susceptibility, biofilm formation, the adherence properties using epithelium cell lines and animal models.

## Methods

### Isolation and growth conditions of *Lactobacillus* strains

Rectal swabs taken from 39 of healthy dogs were examined to isolate *Lactobacillus* strains. In this study, all samples were obtained during routine investigations by practicing veterinarians from veterinary clinics located in two cities of Poland: Krakow and Tarnow. Specimens were transported to the University Centre of Veterinary Medicine, Jagiellonian University – Agricultural University in Krakow at refrigerator temperature (2–8 °C), then plated on MRS agar (Oxoid, UK) and cultured at 37 °C for 48 h in anaerobic conditions (Genbox anaer, bioMerieux, France). The cultivated colonies were identified for genus and species using Gram staining, commercially available API 50CH (bioMerieux, France) system and PCR assays (primers and protocols listed in Table 5). In the case of ambiguous species identification results, MALDI-TOF mass spectrometry (MS) was additionally used.

**Table 5:** Primer sequences used for this study

Microorganism	Primer name	Sequence (5' to 3')	Amplicon size (bp)	Reference
*Campylobacter spp.*	cadF cadR	TTG AAG GTA ATT TAG ATA TG CTA ATA CCT AAA GTT GAA AC	400	[41]
*Campylobacter jejuni*	CJF CJR	ACT TCT TTA TTG CTT GCT GC GCC ACA ACA AGT AAA GAA GC	323	[42]
*Campylobacter upsaliensis*	CUF CUR	AAT TGA AAC TCT TGC TAT CC TCA TAC ATT TTA CCC GAG CT	204	[42]
*Lactobacillus spp.*	LbLMA-1	CTC AAA ACT AAA CAA AGT TTC	250	[43]
	R-161	CTT GTA CAC ACC GCC CGT CA		
*Lactobacillus brevis*	LbrevF LbrevR	CTT GCA CTG ATT TTA ACA GGG CGG TGT GTA CAA GGC	1340	[44]
*Lactobacillus casei—*group	IDL11F IDL03R	TGG TCG GCA GAG TAA CTG TTG TCG CCA CCT TCC TCC GGT TTG TCA	727	[45]
*Lactobacillus crispatus*	Cri 16SI Cri 16SII	GTA ATG ACG TTA GGA AAG CG ACT ACC AGG GTA TCT AAT CC	734	[46]
*Lactobacillus delbrueckii*	IDL31F IDL03R	CCA CCT TCC TCC GGT TTG TCA CTG TGC TAC ACC TAG AGA TAG GTG G	184	[45]
*Lactobacillus rhamnosus*	PrI	CAG ACT GAA AGT CTG ACG G	190	[46]
	RhaII	GCG ATG CGA ATT TCT ATT ATT		
*Lactobacillus salivarius*	Lsal-1 Lsal-2	AAT CGC TAA ACT CAT AAC CT CAC TCT CTT TGG CTA ATC TT	411	[47]

### Isolation and growth conditions of *Campylobacter* strains

Rectal swabs isolated from 19 dogs with diarrhea were examined for *Campylobacter* species. The samples were plated on *Campylobacter* blood-free selective agar CCDA (Oxoid, UK) and cultured for 48 h at 42 ± 1 °C under microaerophilic conditions (Genbox microaer, bioMerieux, France). Species identification of the grown colonies was performed using a commercial, standardized system for the identification of *Campylobacter*—API Campy (bioMerieux, France) and PCR technique, performed according to protocols described previously (Table 5).

### Anti-*Campylobacter* activity of *Lactobacillus* – agar slab method

In total, 30 strains of *Lactobacillus* were collected. All isolates were suspended in 0.9% NaCl so that the optical density (OD) of the suspension at 600 nm was 0.5. The suspensions were seeded onto MRS agar and incubated for 24 h at 37 °C in anaerobic conditions. Then, agar slabs were cut (9 mm in diameter) and placed on *Campylobacter* blood-free selective agar CCDA inoculated with 0.5 ml of the *Campylobacter* indicator strain suspended in 0.9% NaCl (OD600 = 0.5). As indicator strains, two reference *Campylobacter* strains were used: *C. jejuni* ATCC 29,428 and *C. coli* ATCC 33,559. The plates were incubated at 42 ± 1 °C for 48 h in microaerophilic conditions. After incubation, the diameter of the zone of growth inhibition was measured. The experiment was performed in duplicate.

### Anti-*Campylobacter* activity of *Lactobacillus*– serial tenfold dilution method

The current and the subsequent experiments were carried out on four selected *Lactobacillus* strains that had induced significant growth inhibition zones in the agar slab method: 25 K/L/1 (*L. salivarius*), 42 K/L/2 (*L. rhamnosus*), 50 K/L/1 (*L. sakei*) and 55 K/L/1 (*L. agilis*). Four strains of *Campylobacter* genus that had been isolated in this study from faeces of dogs with diarrhea: 18 K/C/1 (*C. jejuni*), 25 K/C/1 (*C. jejuni*), 27 K/C/1 (*C. jejuni*) and 28 K/C/1 (*C. upsaliensis*) were used as target strains to test the inhibitory activity of selected *Lactobacillus*.

Tested *Lactobacillus* strains were cultured in 10 ml of TSB (Tryptic Soy Broth, Biocorp, Poland) for 24 h at 37 °C under anaerobic condition to get a final concentration of 10^7^ CFU/ml (colony-forming units per milliliter). Then, the suspensions were centrifuged (12,000 × *g,* 15 min, 20 °C) and the supernatants were sterile-filtered using a 0.22 μm Millipore filter (VWR, Germany). Strains of *Campylobacter* were also cultured in 10 ml of TSB for 24 h, at 42 ± 1 °C under microaerophilic condition. The final concentration of suspensions was 10^7^ CFU/ml as well. The supernatants of *Lactobacillus* strains and the suspensions of *Campylobacter* cultures were mixed in the ratio of 9:1. The mixtures of *Lactobacillus* supernatants and *Campylobacter* cultures were spread over Columbia Agar with 5% sheep blood (Biocorp, Poland) in the following dilutions: 0, -2 and –4, respectively within 10 min., 8 h and 24 h after the moment they had been mixed. The plates were incubated for 24 h under microaerophilic conditions at 42 ± 1 °C. Campylobacter colonies were enumerated and expressed as CFU/ml. In this experiment 16 samples wereanalyzed in duplicate.

### Anti-*Campylobacter* activity of *Lactobacillus* – well diffusion method

This experiment was performed to determine the mechanism of the antimicrobial activity of selected *Lactobacillus* strains. Isolates were grown in 10 ml of MRS broth for 24 h at 37 °C in anaerobic conditions to get the final concentration of 10^7^ CFU/ml. The suspensions were centrifuged (12,000 × g, 15 min, 20 °C) and the supernatants were sterile-filtered using a 0.22 μm Millipore filter. Then, each sample of supernatant was divided into 2 equal volumes. In half of the samples the pH was adjusted to 6.8—7.0 using 1 M NaOH (to eliminate the influence of organic acids and low pH).

Strains of *Campylobacter* were cultured in 10 ml of TSB for 24 h at 42 °C under microaerophilic conditions and inoculated on Columbia Agar with 5% sheep blood. Then, agar slabs were cut (9 mm in diameter) and filled with 200 ul of the supernatants with neutralized acids, as well as native acidified supernatants (control). After 48 h of incubation at 42 ± 1 °C under microaerophilic conditions, the plates were checked for inhibition zones. The experiment involving 16 samples was run in duplicate.

### Production of H_2_O_2_

The ability of selected *Lactobacillus* strains to produce H_2_O_2_ was determined by semiquantitative Peroxide Test Strip method (Merck, Germany). For this assay, *Lactobacillus* strains were cultured in 10 ml of MRS broth for 24 h at 37 °C in aerobic conditions. The mean density of bacteria at the beginning of an experiment was estimated approximately as 1 × 10^6^ CFU/ml. H_2_O_2_ measurement was performed in three-time intervals: 10 min., 4 h, and 24 h after the start of culture. The Peroxide Test Strip indicates the presence of H_2_O_2_ by a color change on an indicator strip. The results were compared to a provided color scale (detection scale between 0 and 100 mg/L). The experiment involving 16 samples was run in duplicate.

### Tolerance for acidic pH

The group of selected *Lactobacillus* strains were grown overnight in MRS broth at 37 °C in anaerobic conditions and then centrifuged (12,000 × *g,* 5 min, 20 °C). Pellets were washed with PBS and resuspended in 1 ml of PBS (the final suspensions had a value of 1 × 10^8^ CFU/ml). 100 ul of the suspensions were added to 900 ul of MRS broth with pH adjusted to 2.0 and 3.0 with 1 M HCl. Moreover 100 ul of the suspensions were added to 900 ul of MRS broth with pH 6.5 (positive control). The bacteria were incubated at 37 °C in anaerobic conditions. Samples were taken after 3 h and the viable number of bacteria were determined by standard serial tenfold dilution method on MRS agar. The survival of bacteria was expressed as a percentage calculated from the logarithms of the number of CFU after 3 h of incubation in environment with pH 2.0 or 3.0 compared to the logarithms of the number of CFU after the same time intervals in an optimal pH environment (6.5). The experiment involving 16 samples was run in duplicate.

### Bile tolerance test

The bile tolerance test of *Lactobacillus* isolates was performed by addition of bile salts (Merck, Germany) to MRS medium, to the final concentration of bile salts 0.5% and 1% and then the mixture was inoculated with bacteria for 1 × 10^8^ CFU/ml. For the control samples, distilled water was added to MRS medium instead of bile salts. *Lactobacillus* bacteria were incubated in this medium at 37 °C in anaerobic conditions. The samples were collected at time 3 h and to estimate the bacteria survival a standard serial tenfold dilution method on MRS agar was performed. The survival of bacteria was expressed as a percentage calculated from the logarithms of the number of CFU after 3 h of incubation with the addition of bile salts (0.5% or 1%) relative to the logarithms of the number of CFU after the same time intervals without adding the bile salts to the medium. The experiment involving 16 samples was run in duplicate.

### Statistical analysis

All experiments were carried out in duplicate, and the results were expressed as a mean ± standard deviation. To analyze the obtained results, the following statistical tests were used: the one-way analysis of variance (ANOVA) to compare the mean diameters of the inhibition zones for indicator *Campylobacter* strains that were determined to be sensitive to various *Lactobacillus* species and the paired t-test for comparing differences between the counts of *Campylobacter* in serial dilution method after following hours of the experiment. P values lower than 0.05 were considered significant. All calculations were performed using JMP 7.0.2 (SAS, United States) software package.

## Data Availability

The data and materials are available from the corresponding author on reasonable request.

## Abbreviations

C.F.U: Colony Forming Unit
MRS: De Man Rogosa Sharpe
PBS: Phosphate Buffered Saline
PCR: Polymerase Chain Reactin
SD: Standard Deviation
TSB: Tryptic Soy Broth
